# 325. Outcomes of Outpatient Parenteral Antimicrobial Therapy in the Solid Organ Transplant Population

**DOI:** 10.1093/ofid/ofad500.396

**Published:** 2023-11-27

**Authors:** William P Dillon, Al Muthanna Shadid, Austin Parsons, Megan Hardy, Jonathan D Williams, Jennifer McCorquodale, Mayur Ramesh, George J Alangaden

**Affiliations:** Henry Ford Hospital, Detroit, Michigan; Henry Ford Hospital, Detroit, Michigan; Henry Ford Hospital, Detroit, Michigan; Henry Ford Hospital, Detroit, Michigan; Henry Ford Health System, Detroit, Michigan; Henry Ford Hospital, Detroit, Michigan; Henry Ford Hospital, Detroit, Michigan; Henry Ford Health, Detroit, Michigan

## Abstract

**Background:**

Outpatient parenteral antimicrobial therapy (OPAT) provides an effective and convenient means to complete extended courses of antimicrobial therapy for the treatment of serious infections. There is scant data addressing OPAT related outcomes such as readmission in solid organ transplant (SOT) recipients.

**Methods:**

In this observational cohort study, we analyzed all adult SOT recipients discharged from Henry Ford Hospital – an 877 bed quaternary care center in Detroit, Michigan - on OPAT between January 2015 and December 2020. The primary endpoint was 30-day all-cause readmission. The secondary endpoints included evaluation of risk factors associated with readmission (transplant type, reason for OPAT, OPAT related complications, length of treatment, length of stay, discharge disposition, and adequacy of infectious disease follow up and laboratory monitoring) and all-cause mortality at one year.

**Results:**

There were 201 patients discharged on OPAT. Demographics between study populations were comparable (Table 1). A total of 83 out of 201 (41.3%) patients were readmitted. There were 38 (18.9%) patients readmitted for OPAT related complications and 45 (22.4%) for non-OPAT related reasons. Intestinal and multi-visceral transplants were associated with readmission (*p*=0.04 and *p*=0.02 respectively) while renal transplants were protective against readmission (*p*=0.02) (Table 1). Other factors associated with readmission include development of an OPAT related complication including treatment failure (*p*< 0.001) (Table 2). Patients discharged without Infectious Disease follow up were less likely to be readmitted (*p*=0.04) (Table 3) as these patients generally had less serious infections not meriting a follow up appointment. There was no difference in mortality at one year between study populations (Table 3).

**Figure:**
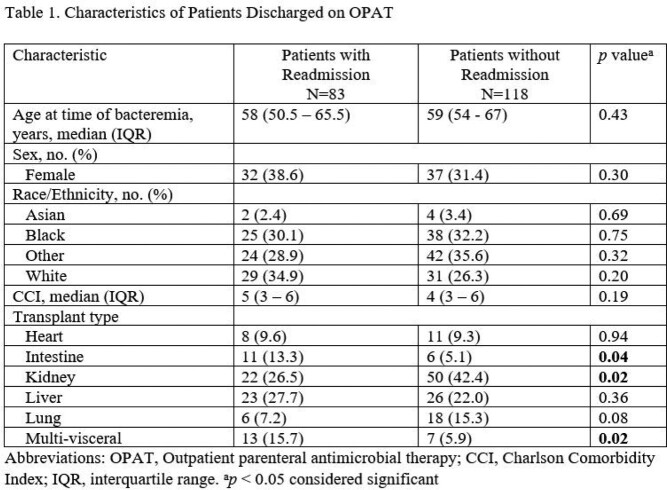

**Figure:**
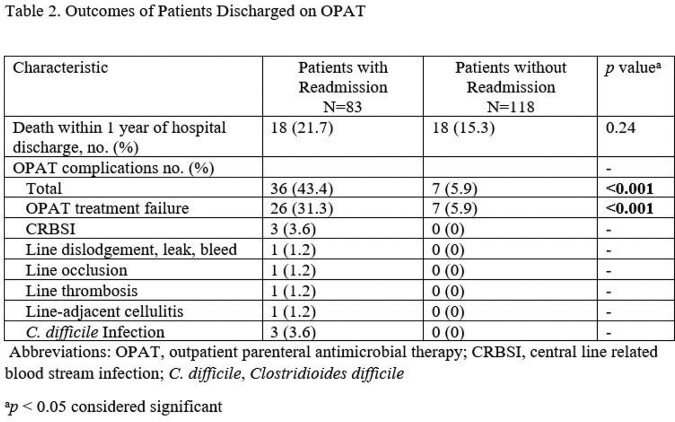

**Figure:**
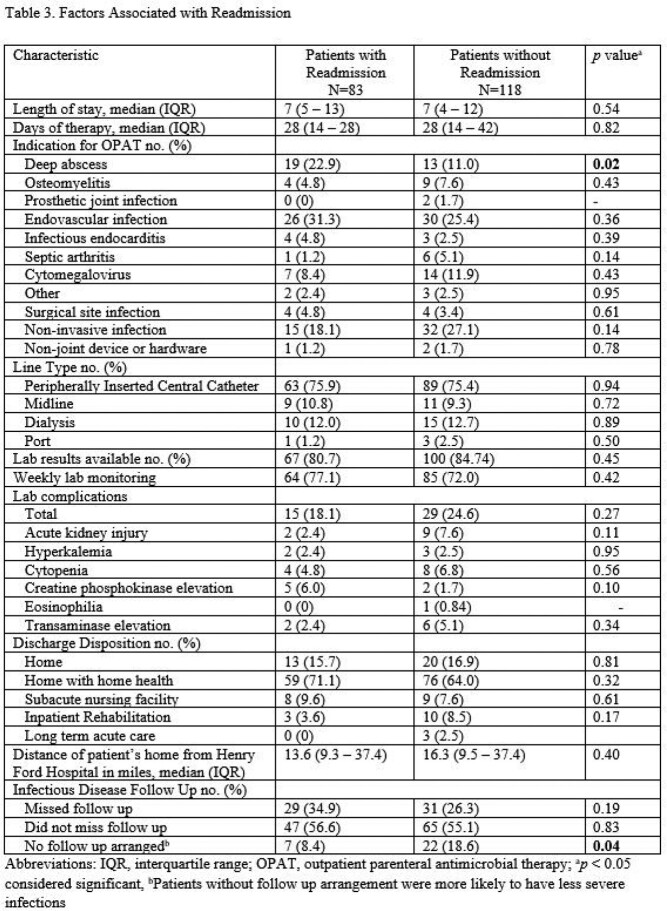

**Conclusion:**

While the overall readmission rate of SOT discharged on OPAT is high, most readmissions were unrelated to OPAT. Patients with readmissions had higher rates of OPAT related complications and treatment failures. Further studies are warranted to optimize OPAT outcomes in the SOT population.

**Disclosures:**

**All Authors**: No reported disclosures

